# A lightweight transformer framework for open set anomaly segmentation in smart city applications

**DOI:** 10.1038/s41598-025-28708-w

**Published:** 2025-11-27

**Authors:** M. Manicka Prabha, K. Suganthi

**Affiliations:** 1 School of Computer Science and Engineering, Vellore Institute of Technology, Chennai, Tamil Nadu 600127 India; 2 Centre for Cyber Physical Systems (CCPS), Vellore Institute of Technology, Chennai, Tamil Nadu 600127 India

**Keywords:** Open-set segmentation, Anomaly segmentation, Lightweight transformer, Smart cities, Computational efficiency, Context-aware segmentation, Engineering, Mathematics and computing

## Abstract

Open-set anomaly segmentation task in diverse infrastructure faces substantial challenges due to its computational overhead and accuracy measures. Although the existing transformer-based methods are efficient, that are limited in the factors of Computational efficiency and accuracy trade-offs. This paper presents LightMask, a lightweight transformer-based architecture designed for efficient, context-aware segmentation of anomalous regions in complex urban environments. The proposed framework has five key contributions: optimized EfficientNet-B0 backbone, adaptive inference mechanism, separable self-attention (SSA) with linear complexity, progressive multi-scale decoder with dynamic early termination, and boundary-aware contrastive loss for open-set anomaly segmentation tasks. LightMask focuses on the lightweight framework with computational efficiency first, while preserving the performance of anomaly detection. The evaluation results showcase that LightMask produces lower parameter count of 4.29 million (16.35 MB) ensures the lightweight structure and a computational efficiency with only 8.72 GFLOPs. For training and evaluation, the Cityscapes and RoadAnomaly datasets were used and the finding reveals the model robustness with 91.79% precision, 93% recall, 77.66% F1 score, 88.28% AUC-ROC, and a low false positive rate of 36.24% at 95% TPR. Based on these findings LightMask balances computational costs with robust anomaly detection capabilities.

## Introduction

The rapid growth of urbanization and the increased use of smart city systems have substantially changed urban surveillance and monitoring systems. As a result, many urban environments are now utilizing state-of-the-art computer vision technologies to enhance public safety and traffic flow through the use of video cameras as well as other smart city systems, and to monitor and protect critical infrastructure. However, the deployment of smart surveillance systems in urban infrastructure provides both computational and accuracy challenges due to the variety of unexpected items and events that may occur in an urban environment that fall outside the boundaries of pre-trained knowledge based category models^1,2^. Therefore, it is very important for smart city systems to be able to recognize and identify unknown objects in real time.

Unlike traditional closed set approaches, open set anomaly segmentation task allows systems to identify and segment objects that were not seen during the training phase^3,4^. This type of resilience is essential in urban settings where unexpected occurrences of objects and possible security threats may appear suddenly. While these models typically work extremely well with established categories of objects. But it often fails intensely when exposed to out-of-distribution (OOD) data, that may lead to misclassifications and a degradation in the reliability of the overall system^5,6^. Especially in the context of smart cities, there may be a major impact when the system fails to detect the anomalies. So these challenges needs to be addressed in the urban infrastructure and public safety systems.

A primary challenge in smart cities is designing lightweight anomaly segmentation systems with low computational overhead in terms of memory. Most current state-of-the-art models require significant computational resources and therefore cannot be deployed on resource constrained edge devices^7,8^. This is a major challenge in using these models in an urban application is managing the inherent trade-off between computational efficiency and model performance resulting from the need for both real-time processing and segmentation accuracy.

The recent innovations in the transformer architecture have shown an impressive competency to capture contextual information and long-range dependencies between contextual elements, making it well-suited to understand complex urban scenes^9,10^. Nonetheless, the quadratic time complexity of the standard self-attention mechanism poses significant key constraints on its use for real-time applications, with very less access to computational resources^11,12^. Therefore, the development of new lightweight transformer models able to maintain the ability of contextual reasoning and having a linear time complexity, represents a critical research direction for the use in smart city applications.

The introduction of adaptive inference processes is also a necessity for effective outlier segmentation systems for urban environments. The complexity of the scene, the level of lighting conditions, and the presence of anomalous objects all noticeably affect the computational requirements for the analysis of different urban areas^9,13^. Recent advances in vision tasks have shown that real-time sped capabilities may be realized by means of streaming architectures which permit the incremental processing of sequential frames without requiring knowledge of the whole set of sequences^14^. Such methods have achieved success in video captioning, but the principles of incremental processing and memory compression are easily susceptible to real-time outlier detection, where the prompt detection of outlier patterns is of great importance. It is valid to mention that conventional techniques which may apply to each of the inputs approximately the same computational costs result in a less efficient allocation of the resources available and a low efficiency in the working of the system as a whole. The employable qualities of the anomaly segmentation systems may thus be enhanced by the development of dynamic computation techniques which can adaptively vary the processing power required in accordance with the complexity of the input.

LightMask addresses these important issues mentioned above through its innovative design as a novel, lightweight, transformer-based model, that is particularly suited to be applied to anomaly object context aware segmentation on highly complex urban visual scenes. The key architectural innovations of LightMask have been described as follows:

1. **Lightweight Backbone layer:** To reduce the computational overhead, the lightweight backbone like EfficientNet-B0 has been used. This layer extracts the high level features using the pre-trained CNN.
2. **SSA Mechanism:** This module decomposes the attention process with linear complexity.
3. **Progressive Decoding Strategy:** This component refines the output/features in multiple stages. Using the early termination based on the image complexity.
4. **Adaptive Inference Framework:** This layer adjusts the computational path based on the image complexity in dynamic initialization.
5. **a region-level objectness branch and boundary-aware contrastive loss:** This block explicitly focuses on enhancing the precision for boosting anomaly localization and boundary precision in open-set segmentation.

The system was designed with the purpose of developing a lightweight framework with low computational costs, but also maintain the same level of accuracy required for the segmentation systems. Through extensive experimental evaluation on standard benchmarks, including Cityscapes^15^, for structured scene segmentation, and the Road Anomaly Dataset^16^ for anomaly segmentation, we demonstrate that LightMask achieves superior efficiency-accuracy trade-offs compared to existing approaches. Our results show significant improvements in inference speed while maintaining or improving segmentation accuracy across diverse environmental conditions and anomalous categories.

The remainder of this paper is organized as follows: Section “Related works” provides a comprehensive review of related work in open-set recognition and anomaly segmentation, transformer-based segmentation architectures, Efficient Segmentation and lightweight design, adaptive inference and progressive processing and Research gap and our contributions. Section “Methodology” details the LightMask architecture, including our novel contributions in separable attention, progressive decoding, adaptive inference, and objectness head and boundary-aware contrastive loss. Section "Experimental design and comprehensive evaluation" presents our experimental methodology, comprehensive evaluation results and Discussions. Finally, Section “Conclusion” concludes with a summary of our contributions and their significance for smart city applications.

## Related works

Open-set anomaly segmentation in urban environments have key challenges that requires efficient models that balance between detection capabilities and computational efficiency. The literature review has been carried out with four key dimensions. Those are anomaly detection paradigms, transformer-based segmentation architectures, efficiency prioritized design methodologies, and the research gap exists in the open-set anomaly segmentation problems.

### Open-set recognition and anomaly segmentation

Traditional semantic segmentation techniques operate with closed-world assumptions, limiting its applicability to real-world scenarios with unexpected objects^3,18^. Open-set Semantic Segmentation (OSS) identifies the known and unknown object categories^18^, with key challenges in identifying Unknown Unknown Classes (UUCs) due to the spatial correlations in pixel-wise tasks.

Anomaly segmentation methods have evolved through several approaches like reconstruction-based methods^16^, outlier exposure^17^, and uncertainty-aware domain adaptation^19^. Recent work by Nguyen et al.^20^ introduces DifferNet for automatic labeling in unfamiliar road environments, while MiniMaxAD^21^ employs large kernel convolutions and autoencoders for feature-rich anomaly detection. Mask2Anomaly^22^ and MaskFormer^23^ demonstrates the effectiveness of mask-based classification for reducing false positives in unknown object localization.

Even though with these advanced technologies, existing anomaly segmentation models encounters two key challenges: (1) computational demands, and (2) inability to adapt inference complexity dynamically based on scene characteristics. These drawbacks motivate our efficiency first design approach.

### Transformer-based segmentation architectures

Vision Transformers evolved by the ability to model a scene with global contextual modeling has enabled the development of semantic segmentation models^6,9,12^. For example, SETR^13^, and SegFormer^24^ provide strong performance of transformer encoders as well as hierarchical approaches such as those found in Swin Transformer^7^ to improve efficiency.

Although these approaches have shown great promise, one of the main limitations they face is that traditional multi-head self-attention (MHA) has a quadratic time complexity ($O(N^2)$). As a result of this limitation, we see that the standard SegFormer architecture uses 744.8 GFLOPS; while an efficient variant called RTLinearFormer consumes over 100 GFLOPS^25^. This level of computational demand makes it difficult to apply these architectures where there are very low latency requirements and/or very limited power budgets.

As a means of simplifying transformers there are now various forms of linear attention mechanisms^26^, as well as separable self-attention mechanisms^27^. Each approach achieves *O*(*N*) complexity, but maintains representation capabilities. These innovational architectures show that it is possible to make transformer-based image segmentation approaches computationally reasonable, but these have not yet been implemented in an open-set anomaly detection context.

### Efficient segmentation and lightweight design

As researchers have recognized the computational burden associated with large, heavy architectures they have started to explore how to create models that are lighter than their predecessors. As such, researchers have developed efficient architectures by using compound scaling methods^10^, as well as by reducing the size of models through deep compression techniques^5^, such as pruning, quantization, and knowledge distillation.

In the area of semantic segmentation light-weight architectures such as DeepLabV3+^28^, PSPNet^29^ and SegNet^30^ are also based on efficient encoder decoder architectures. Recently, new work by Mazhar et al^31^ introduces IRDP-Net, a light-weight architecture for road scene segmentation employing inverted residuals and dilate pyramids with a minimal number of parameters on higher precision. MobileViT^32^ considers a new domain of vision transformers with improved efficiency on mobile scale.

The unique challenges of open set anomaly detection and specifically, the challenge of high recall in safety critical anomaly detection but maintaining precision is not resolved in terms of in computation resources.

### Adaptive inference and progressive processing

Beyond statistical architectural efficiency, adaptive inference mechanisms enable dynamic computation based on input complexity. Early-exit strategies^33^ allow networks to terminate processing when confident predictions are reached. Context-aware segmentation methods^34^ leverage spatial priors to improve accuracy, though typically with fixed computational costs.

Progressive decoding strategies^35^ refine predictions through multi-scale processing, enabling coarse-to-fine segmentation. However, existing approaches lack mechanisms to modulate decoding depth based on scene characteristics, resulting in unnecessary computation for simple inputs.

### Research gap and contribution

Current literature reveals a critical gap with no existing work simultaneously addresses open-set anomaly segmentation with extreme computational efficiency. Specifically, Transformer-based segmentation methods^13,24,25^ achieve strong performance but require 100–700 + GFLOPs, Lightweight segmentation networks^28,31,32^ are efficient but designed for closed-set tasks, Anomaly detection methods^20–22^ either lack real-time capability or require substantial computation.

The proposed LightMask architecture addresses these limitations through a comprehensive approach that integrates efficient backbone networks, separable attention mechanisms, progressive decoding strategies, and adaptive inference frameworks and Boundary-aware contrastive loss with region-level objectness for improved open-set generalization.

## Methodology

### Overview of LightMask architecture

This section describes a lightweight transformer-based architecture designed to support efficient open-set segmentation of anomalous objects in context of urban roadways. The proposed framework LightMask, solves the most important challenges of anomaly segmentation in smart-cities applications, simultaneously maintaining the computational efficiency and achieving high levels of accuracy. LightMask has five main components: reliable backbone network, SSA mechanisms, objectness guidance at region level, progressive decoding with early termination, and adaptive inference with image quality complexity estimation.

**Fig. 1:**
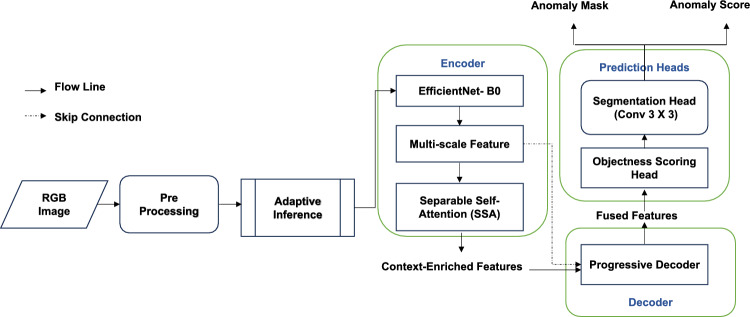
Overview of the proposed LightMask architecture.

A complete architecture is depicted in Fig. 1 and it uses an encoder decoder paradigm with an integrated attention enhanced feature extraction. This approach combines dynamic computational algorithms, multi-scale integration of features, and a novel design of an attention-mechanism so that a slim architecture can be enabled with lower computational rates, and the benchmark at the same time similar accuracy in segmenting anomalies can be maintained.

### Efficient backbone architecture

#### Backbone selection and modification

A backbone network acts as a cornerstone of the feature extraction of the LightMask models. The EfficientNet-B0^10^ used as a backbone that offers the best trade-off among accuracy and efficiency of computing measures. The architecture has an efficient parameter count of around 4.3million parameters which allow the architecture to achieve excellent performance by the use of the scaling of the compounds.

The modification of the backbone encompasses:

1. **Classifier Removal:**To change the network for dense prediction tasks, the final classification layer is eliminated.
2. **Feature Extraction Focus:**To extract hierarchical features, deliberately place hooks at different sizes.
3. **Output Dimension Adaptation:** Four distinct scales with dimensions [24, 80, 192, 1280] channels are applied to extract feature maps.

Given an input RGB image $I \in \mathbb {R}^{H\times W\times 3}$, multi-scale features are extracted as:

1 $$\begin{aligned} \boldsymbol{F}_{\boldsymbol{i}} = {\textbf {Extract}}\left( \boldsymbol{I,} {\textbf {Scale}}_{\boldsymbol{i}}\right) \quad {\textbf {where}} \quad \boldsymbol{i \in \{1,2,3,4\}.} \end{aligned}$$

where $F_i$ denotes the feature map at scale *i* with gradually increasing semantic richness and decreasing spatial resolution.

### Separable self-attention mechanism

#### Attention complexity reduction

Conventional self-attention methods are computationally expensive for high-resolution feature maps due to their quadratic complexity $O(N^2)$^37^ for sequence length N. The primary limitation arises while estimating the Pairwise attention weights between all spatial positions in the feature tensor. In the instance of an input feature map $X \in \mathbb {R}^{B\times C\times H\times W}$, the spatial dimensions are adjusted into a sequence of length $N = H \times W$ using standard self-attention, which involves the computation of an attention matrix $A \in \mathbb {R}^{N \times N}$. This significantly limits the usage in high-resolution computer vision tasks due to the memory consumption and computational cost that scale quadratically with input resolution. A difficult scaling characteristic that limits realistic deployment scenarios is caused by the requirement to run query-key dot products over all $N^2$ position pairs, followed by weighted value aggregation and Softmax normalization. These steps further increase the computing burden.

To overcome this hurdle, we propose a novel SSA mechanism that accomplishes linear complexity *O*(*N*) by collapsing the attention computation into spatial and channel components. The SSA mechanism works by applying spatial attention (As) and channel attention (Ac) processes^38^ in a factorized manner. Given input features $X \in \mathbb {R}^{B\times C\times H\times W}$, the spatial attention component generates spatially-refined features with complexity $O (N \times C)$ by independently computing attention weights for each channel dimension. The spatially-attended features are exposed to the subsequent channel attention, which preserves inter-channel interdependence with complexity $O(C^2)$. The adaptive gating mechanism Gate(X) in the mathematical formulation enables dynamic feature selection and improves cognitive capacity by regulating the attention weights by element-wise multiplication $\odot$. For typical computer vision scenarios where $C \ll N$, this separable decomposition minimizes the overall computational complexity from $O (N^2 \times C)$ to $O (N \times C + C^2)$, which approaches linear scaling *O*(*N*)^26^. This makes it feasible for the efficient processing of high-resolution feature maps while maintaining the expressive power of self-attention mechanisms.

The SSA mechanism serves via the following mathematical formulation:

2 $$\begin{aligned} {\textbf {SSA}}\boldsymbol{(X)} = {\textbf {Gate}}\boldsymbol{(X) \odot } {\textbf {SpatialAtt}}\boldsymbol{(X) + (1 -} {\textbf {Gate}}\boldsymbol{(X)) \odot } {\textbf {ChannelAtt}}\boldsymbol{(X)} \end{aligned}$$

where $X \in \mathbb {R}^{B\times C\times H\times W}$ denotes the input feature map, where *B*, *C*, *H*, and *W* correspond to the batch size, number of channels, height, and width, respectively. The operator $\odot$ signifies element-wise (Hadamard) multiplication, and Gate(X) denotes an adaptive gating mechanism applied to the feature map *X*.

#### Spatial attention component

If high-resolution feature maps are concerned, the spatial attention sub-module is also able to capture rich spatial dependencies at a long-range of scales while maintaining computational tractability.Given a feature map $X \in \mathbb {R}^{H\times W\times C}$ extracted from the encoder, we initially apply a point-wise convolution to reduce the channel dimensionality.

3 $$\begin{aligned} \boldsymbol{X}_{{\textbf {reduced}}} = {\textbf {Conv}}_{\boldsymbol{1\times 1}}\boldsymbol{(X)} \end{aligned}$$

Before performing the attention technique, this block drastically reduces the number of parameters and memory consumption by shrinking the number of channels from *C* to *C*/2. Standard multi-head self-attention can then be applied adequately by reshaping the reduced feature map into a series of spatial tokens of shape $(H \cdot W) \times C/2$:

4 $$\begin{aligned} {\textbf {SpatialAtt}}\boldsymbol{(X) =} {\textbf {MultiHeadAtt}}\boldsymbol{(} {\textbf {Reshape}}\boldsymbol{(}\boldsymbol{X}_{{\textbf {reduced}}}\boldsymbol{))} \end{aligned}$$

The classical dot-product attention is used in this approach to calculate the attention weights:

5 $$\begin{aligned} {\textbf {Attention}}\boldsymbol{(Q, K, V) =} {\textbf {softmax}}\boldsymbol{( \frac{Q K^\top }{\sqrt{d_k}} ) V} \end{aligned}$$

where ${Q, K, V} \in \mathbb {R}^{(H \cdot W) \times d_k}$ are obtained from the flattened spatial domain and comprise the query, key, and value matrices, respectively. This method eliminates the entire quadratic cost that generally comes with traditional self-attention techniques and enabling the model to simulate spatial correlations across distant pixels.

**Fig. 2:**
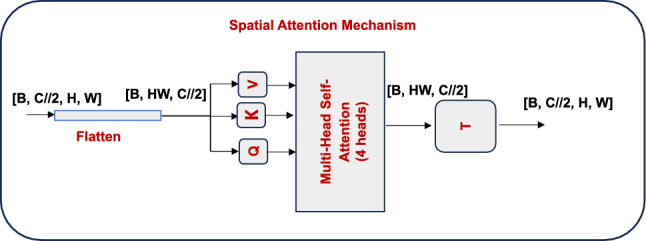
Structure of Spatial attention mechanism.

The spatial attention mechanism, as shown in Fig. 2, captures contextual dependencies between spatial locations. The input tensor [*B*, *C*/2, *H*, *W*] is flattened to produce a sequence of spatial tokens [*B*, *HW*, *C*/2]. These tokens serve as input to a 4-head self-attention module, where separate *Q*, *K*, *andV* projections are learned. The attention-enhanced output sequence is reshaped and transposed back to its original spatial form [*B*, *C*/2, *H*, *W*]. This intermediate output then passes through the Upsample + Pad block, which performs zero-padding along the channel dimension (from *C*/2 to *C* to ensure the dimensional alignment with the Channel Attention output for the final adaptive fusion.

#### Channel attention component

The channel attention component is intended to capture cross-channel dependencies in a lightweight but descriptive way to enhance spatial modeling. We originate with Global Average Pooling (GAP) to compress the spatial dimensions of an input feature map $X \in \mathbb {R}^{H\times W\times C}$, resulting in an efficient channel descriptor $\in \mathbb {R}^{C}$. After passing this descriptor via a two-layer bottleneck composed of two $1\times 1$ convolutions with a ReLU non-linearity in between, followed by a sigmoid activation function:

6 $$\begin{aligned} {\textbf {ChannelAtt}}\boldsymbol{(X) = X \odot \sigma (} {\textbf {Conv}}_{\boldsymbol{1\times 1}}\boldsymbol{(} {\textbf {ReLU}}\boldsymbol{(} {\textbf {Conv}}_{\boldsymbol{1\times 1}}\boldsymbol{(}\text {GAP}\boldsymbol{(X)))}\boldsymbol{)}\boldsymbol{)} \end{aligned}$$

Here, channel-wise multiplication is indicated by $\odot$, and gating of specific channels is made possible by $\sigma$, $\sigma$ which guaranties that the output weights fall within the range [0, 1]. With a complexity of $O(C^2/r)$, where *r* is the reduction ratio (set to 16), this computation can be done efficiently even with high-dimensional channel spaces.

**Fig. 3:**
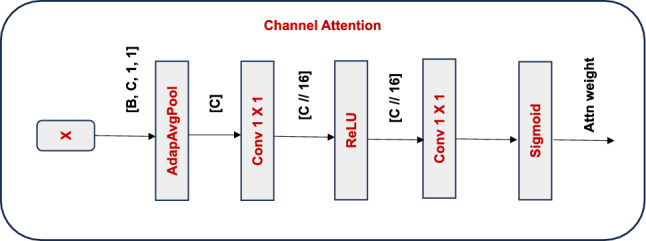
Structure of the Channel Attention mechanism.

The channel attention mechanism, as shown in Fig. 3, emphasizes informative channels by first compressing spatial information through adaptive average pooling, which produces a channel descriptor [*B*, *C*, 1, 1]. The result is passed through a bottleneck structure using 1×1 convolutions and a non-linearity to first reduce the channel dimensionality to *C*/16 and subsequently restore the dimensionality back to *C*. A final sigmoid activation produces channel-wise attention weights [*B*, *C*, 1, 1] that are then spatially transmitted to modulate element-wise modulation of the original feature map, producing the final channel-attended feature map [*B*, *C*, *H*, *W*].

#### Adaptive gating mechanism

We propose an adaptive gating mechanism that adjusts the relative relevance of each branch based on the input features to dynamically balance the contributions from spatial and channel attention paths. In particular, GAP is utilized for generating a global representation of the input, which then undergoes a sigmoid activation and a linear transformation:

7 $$\begin{aligned} {\textbf {Gate}}\boldsymbol{(X) = \sigma }\boldsymbol{(} {\textbf {Linear}}\boldsymbol{(} {\textbf {GAP}}\boldsymbol{(X)))} \end{aligned}$$

**Fig. 4:**
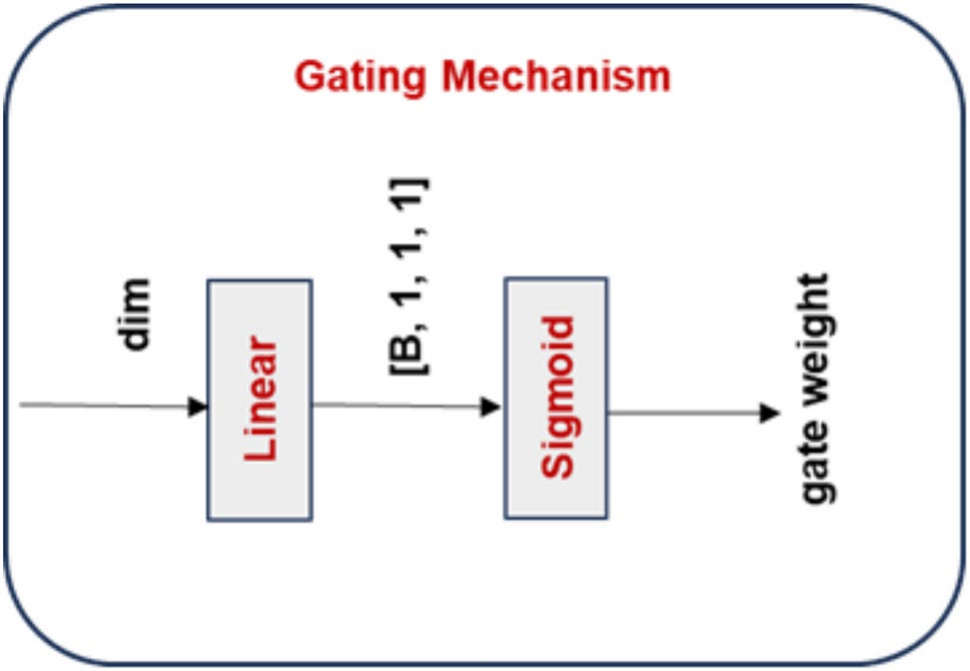
Structure of the Gating mechanism.

The gate mechanism in Fig. 4 learns a dynamic scalar weight from the globally pooled feature representation using a lightweight linear layer followed by a sigmoid activation. This gate weight adaptively balances the contribution of spatial and channel attention during the final feature fusion stage.

By acting as an adaptive weighting coefficient, the scalar gate output leads the model to prioritize channel attention in circumstances where inter-channel feature interactions predominate (such as semantic or object-level representations) as well as spatial attention in instances where spatial structures (such as edges or textures) are prominent. This gating mechanism adds to the model the generalization properties in a wide variety of scene situations, which allows a flexible and content aware synthesis of the two modalities of attention.

**Fig. 5:**
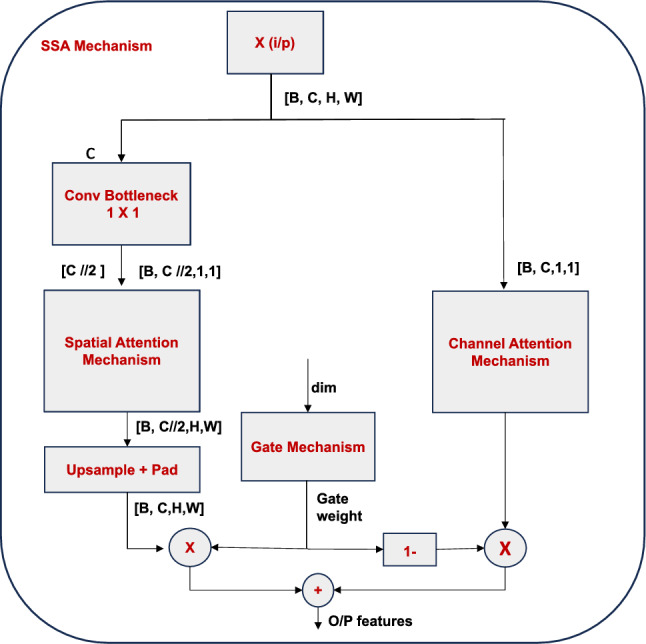
Illustration of SSA mechanism.

Figure 5 gives a schematic figure of the Separable Self-Attention module, where spatial and channel attentions are calculated separately. A dilution step of gating protocol then combines the responses of the two streams of attention to produce context-enhanced feature representations. This structure significantly lowers the computational load with an expressive capacity.

### Progressive decoding with early termination

#### Multi-scale decoder architecture

The proposed decoder uses a progressive multi scale decoding approach as described in^35^, where segmentation masks are progressively refined at a hierarchy of spatial resolutions in order to support efficient and flexible semantic prediction. By increasing the granularity of segmentation, this multilevel refinement also allows for the possibility of early termination, allowing the network to stop the computation for the simple instances in the middle, continued by conserving the computational resource in the inference stage.

The decoder is built up from four sequential decoder modules associated with increasing feature resolutions, represented as $D_i$, for $i \in \{1, 2, 3, 4\}$. Leveraging both the spatial accuracy of low-level feature maps and the semantic accuracy of higher-level representations, these modules are designed in a top-down structure. The formal definition of the decoding protocol is given below:

8 $$\begin{aligned} \boldsymbol{D_i} = {\textbf {DecoderBlock}}\boldsymbol{(F_i, D_{i-1}), \quad D_0 = \emptyset } \end{aligned}$$

where $F_i$ is the encoded feature map at scale *i*, and $D_{i-1}$ is the upsampled decoder output from the preceding coarser scale. The segmentation technique results in coarse-to-fine segmentation masks with structural coherence. The progressive architecture is especially beneficial for simple and for complex scenes, in which the output in the early stage can be sufficient to be exited, and in which the object boundary delineation is further improved in each iteration.

#### Decoder block design

**Fig. 6:**
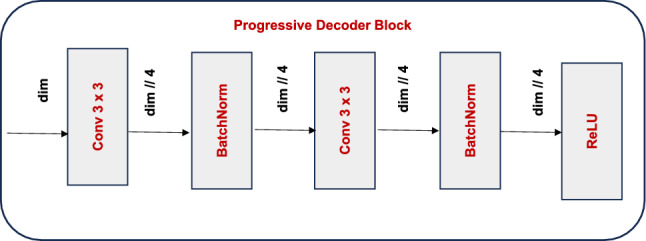
Progressive Decoder Block Architecture.

Each Decoder Block is deliberated as lightweight yet sufficiently demonstrative, owning a bottleneck architecture that diminishes feature dimensionality while preserving critical semantic information. The design depicted in Fig. 6 was initiated by shrinking the input feature map $F_i$ through a $3 \times 3$ convolutional layer that reduces the number of channels to one-fourth of the original dimension. This reduction enhances parameter efficiency by reducing the computational effort and serves as a channel bottleneck. The output is passed via a Batch Normalization (BN) layer and a ReLU non-linearity to maintain learning and introduce non-linearity. A second $3 \times 3$ convolution is applied to refine the features for further extraction. The output captured was then passed through a batch normalization layer and then to ReLU activation function. The resulting refined feature map retains the spatial resolution with more fine-grained semantic information. The decoder module execution flow was represented in the figure. Each decoder block contributes to maintain the accuracy and efficiency of mask refinement with these architectural modifications. This makes the model suitable for smart city applications.

#### Region-level objectness prediction

In the quest for improving the spatial distinction of segmentation predictions especially in complex urban regions identifying anomaly regions, we introduce a Region-Level Objectness Head as an auxiliary supervision branch at each decoder level. More specifically, this component efficiently produces objectness scores right at each spatial location where a value near one indicates a foreground (object-like) area and a value near zero indicates a background.

The Objectness Head is modeled as a very lightweight convolutional sub-network that is attached to each decoder block output $D_i \in \mathbb {R}^{B\times C\times H\times W}$. It consists of a sequence of convolutional operations

9 $$\begin{aligned} {\textbf {ObjectnessHead}}\boldsymbol{(D_i) =} {\textbf {Conv}}_{\boldsymbol{1\times 1}}\boldsymbol{(} {\textbf {ReLU}}\boldsymbol{(} {\textbf {BN}}\boldsymbol{(} {\textbf {Conv}}_{\boldsymbol{3\times 3}}\boldsymbol{(D_i))))} \end{aligned}$$

The implementation encompasses

- A $3\times 3$ convolution performs channel dimensionality reduction from *C* to *C*/2
- Batch Normalization and ReLU activation
- Followed by a $1\times 1$ convolution projecting to a single-channel objectness score map $O_i \in \mathbb {R}^{B\times 1\times H\times W}$.

Unlike the logits applied in semantic segmentation, the objectness-score map can provide the generalized saliency information that helps locate the anomalous regions coarsely especially in the weakly defined contexts.

1. **Foreground Enhancement:** This term adds the confidence to the mask prediction in spatially ambiguous regions, and it works even more efficiently in scenes with objects of small size, or those that are covered.
2. **Auxiliary Regularization:** Auxiliary Regularization applies as a regularizer that enforces object-centric inductive biases across in sequential layers of decoder, improves feature robustness without additional overhead.

#### Feature fusion strategy

To better depict the progressive decoder, feature fusion approach is applied to every decoding stage, so that multi-scale contextual information can be successfully combined. The process plays a pivotal role in both acquiring fine spatial knowledge on smaller scales, and fine semantic knowledge on larger scales. It uses the combination of the previous and current decoder output of the decoding block respectively $D_{i-1}$ and $D_i$ in the fusion process, where $D_{i-1}$ is the output of the previous, coarser decoding block, and $D_i$ represents the features of the current decoder block. The fusion functional is stated to be:

10 $$\begin{aligned} {\textbf {Fusion}}\boldsymbol{(D_i, D_{i-1}) =} {\textbf {Conv}}_{\boldsymbol{1\times 1}}\boldsymbol{(} {\textbf {Concat}}\boldsymbol{(D_{i-1},} {\textbf {Upsample}}\boldsymbol{(D_{i-1})))} \end{aligned}$$

here:

- Upsample refers to a bilinear interpolation function that adjusts $D_{i-1}$ to match the spatial resolution of $D_i$, ensuring alignment for subsequent operations.
- Concat means channel-wise concatenation, based on which the upsampled features, occurring at the unrefined level, are concatenated with the modern decoder output in the channel dimension. Such combination promotes joint inference among semantic cues based on various resolutions.
- The resulting concatenated feature maps are further subjected to a convolutional layer of size $1 \times 1$ that acts as a channel mixer thus allowing a learnt combination of cross-scale information and reducing dimensional redundancy.

This multi-scale fusion hierarchy feature integrates the high-end abstract representations, with the low-end spatial accuracy, and as such produces better segmentation accuracy, especially in the areas of boundaries and fine structures. This lightweight nature of the fusion block also ensures that it has negligible computational cost, which makes it be able to fit the overall efficiency demands of the progressive decoding framework.

#### Confidence-based early termination

In progressive decoding pipeline, we combine a confidence-based early stopping mechanism, to optimize the performance of inference and reduce unnecessary computation with relative inputs. Making sure to evaluate predictive confidence at every decoding level systematically. If the current prediction is sufficient then avoid the additional work of evaluating more predictive confidence by which the computational cost has been reduced.

There is a lightweight prediction head that is applied to the decoder output, denoted as $D_i$ to estimate a scalar confidence score, denoted as $C_i$ is estimated at each decoding level *i*. As an example, in this head, global average pooling (GAP) is used to combine spatial information, and then the result is flattened, and the flattened result is used to create a fully connected layer with sigmoid activation. It is denoted as:

11 $$\begin{aligned} \boldsymbol{C_{i} = \sigma (} {\textbf {Linear}}\boldsymbol{(} {\textbf {Flatten}}\boldsymbol{(} {\textbf {GAP}}\boldsymbol{(D_i))))} \end{aligned}$$

The sigmoid function, which bounds the confidence score in the interval [0, 1], is represented here by $\sigma (\cdot )$. The appropriateness of the current prediction is evaluated using an assumed confidence threshold $\tau = 0.8$. If the confidence score at scale *i* exceeds a particular threshold during inference, the decoding process terminates early, and the mask prediction will be selected as the outcome. The prescribed prerequisite for an early termination is:

12 $$\begin{aligned} {\textbf {Terminate}} = {\left\{ \begin{array}{ll} {\textbf {True}}, & {\textbf {if }} \boldsymbol{C_i> \tau } {\textbf { and not in training mode}}, \\ {\textbf {False}}, & {\textbf {otherwise}}. \end{array}\right. } \end{aligned}$$

This adaptive method enables the model to allocate computation proportionally to input complexity: simple images with coherent semantic regions can be processed at coarser decoding scales, while more intricate scenes are passed via finer-grained stages for enhanced segmentation precision. As a result, the model produces a favorable trade-off between accuracy and efficiency, making it well-suited for deployment in real-time or resource-constrained environments.

### Adaptive inference framework

The paradigm of dynamic computation that has been developed through the Adaptive Inference Framework scales the pathway through which, the model selects to run for the estimated complexity of the input image. The method offers the model to vary attention path, depth of decoding, and feature resolutions based on the level of complexity of the scene to provide the best trade-off between accuracy and efficiency. To ensure efficiency, this process guarantees that simple inputs are computed using the minimum number of resources and complicated situations are computed further.

#### Image complexity estimation

The framework also uses a hybrid image complexity estimation module which combines both learnt and handcrafted metrics to estimate the input difficulty. The gradient-based complexity measure, which is abbreviated as $C_{grad}$, is the sum of first-order image gradients and is used to quantify the average image gradient in both horizontal and vertical directions.

13 $$\begin{aligned} \boldsymbol{C}_{{\textbf {grad}}}\boldsymbol{(I) = \frac{1}{2}} \boldsymbol{(} {\textbf {mean}}\boldsymbol{(} \boldsymbol{\left| \nabla _x I \right| } \boldsymbol{) +} {\textbf {mean}}\boldsymbol{( \left| \nabla _y I \right| )} \boldsymbol{)} \end{aligned}$$

This measure is useful in capturing local intensity variations and edge density which are considered to be indications of visual complexity. Besides that, a trained measure of complexity, denoted as $C_{learned}$, is lifted out by a small neural network architecture named ComplexityNet, that measures scene complexity in a data driven method.

14 $$\begin{aligned} \boldsymbol{C}_{{\textbf {learned}}}\boldsymbol{(I) =} {\textbf {ComplexityNet}}\boldsymbol{(I)} \end{aligned}$$

The model component represents higher-order textural and structural information that might be unseen through gradient information. The final composite complexity score, *C*, may either be the mean of $C_{grad}$ and $C_{learned}$,or a weighted mean learned in the implementation.

After the complexity score is calculated *C*, the system uses three level decision policy to choose the optimal mode of processing.

- The Simple Mode $(C <0.3)$, is to be used in the case of low-complexity inputs. It minimizes the inference cost by rigorously imposing early termination and with sparse attention calculation.
- Medium Mode $(0.\le C<0.7)$, is used as the main mode of progressive decoding and partial attention calculation to ensure efficiency and performance.
- Complex Mode $(C \ge 0.7)$, is used when there is a high complexity in the image. Here, the full decoding is allowed and specific attention provided to allow the best segmentation accuracy.

This policy guarantees the dynamic distribution of computing resources, which match with the semantic and structural content of any image.

#### Dynamic computation adjustment

The model varies with three key elements of computation dynamically depending on the processing mode that is selected.

1. Attention Processing
  To minimize the computational requirements, Simple Mode only uses the initial two attention layers, thus, excludes further attention layers.
  With the level of complexity, the Medium and Complex modes add further attention module gradually.
2. Decoder Depth
  In Simple Mode, the earliest termination is used more aggressively and, in many cases, termination will happen after the first or second decoder block.
  Decoding is performed in Complex Mode where every step is completely run to aid in refining the mask comprehensively.
3. Feature Resolution
  In low-complexity images, can run feature maps at lower-resolution to make inference faster.
  Full-resolution processing is also set to Complex Mode, so that complex scenes are not performed with reduced spatial detail.

The key component of inference strategy was obtained using the adaptive computation estimation. That enables model efficiency without compromising the performance based on the image complexity.

### Loss function design

During training three key components of loss function were used to enhance the efficient learning at multiple scales and complex image patterns. These components were the primary segmentation loss that guides the generation of final prediction, then, intermediate supervision done at each decoder stage to perform deep supervision and finally the confidence regularization loss which terminate early stage based on the image complexity.

#### Primary segmentation loss

To effectively address pixel-wise classification reliability and minimize foreground-background class imbalance, the primary segmentation loss is organized as a composite of cross-entropy loss and dice loss^39^.

15 $$\begin{aligned} \boldsymbol{L}_{{\textbf {seg}}}\boldsymbol{ =} \boldsymbol{L}_{{\textbf {CE}}}\boldsymbol{ + L}_{{\textbf {Dice}}} \end{aligned}$$

#### Intermediate supervision loss

At each decoder scale except the final level, intermediate supervision is employed to enhance gradient flow during training and support **multi-scale learning**. At each step of the progressive decoding pipeline, this stimulates the model to produce significant predictions.

16 $$\begin{aligned} \boldsymbol{L}_{{\textbf {inter}}}\boldsymbol{ = \sum _{k=1}^{K-1} w_k} \boldsymbol{( L}_{{\textbf {CE}}}^{(k)} \boldsymbol{+ L}_{{\textbf {Dice}}}^{\boldsymbol{(k)}} \boldsymbol{)} \end{aligned}$$

where *K* is the total number of decoder stages, and $w_k$ represents the weight factor for supervision at stage *k*, analytically set to 0.5. Each intermediate prediction $\hat{y}_k$ is monitored using the same segmentation objective as the final output.

#### Confidence regularization loss

To enhance the reliability of the confidence-based early termination technique, a confidence regularization loss is introduced. This loss strengthens the confidence scores $C_i$ predicted at each decoder stage to slant harmony for correctly predicted masks

17 $$\begin{aligned} \boldsymbol{L}_{{\textbf {conf}}} \boldsymbol{= \sum _{i=1}^{N} \left| C_i - 1 \right| } \end{aligned}$$

Here, *N* represents the number of decoder scales. Minimizing this term supports refining the confidence estimation and supports the adaptive stopping logic during inference.

#### Objectness loss

To supervise the Region-Level Objectness Head, we introduce an Objectness Loss that penalizes misalignment between predicted objectness scores and ground truth foreground presence. Let $O_i \in \mathbb {R}^{B \times 1 \times H \times W}$ be the raw objectness logits predicted by the objectness head, and let $\hat{O}_i = \sigma (O_i) \in [0,1]^{B \times 1 \times H \times W}$ be the final objectness score map after sigmoid activation. We define the objectness loss at each stage as binary cross-entropy (BCE)^39^

18 $$\begin{aligned} \boldsymbol{L}_{{\textbf {obj}}} \boldsymbol{= \frac{1}{N} \sum _{i=1}^{N}} {\textbf {BCE}}\boldsymbol{\left( O_i, G_i \right) } \end{aligned}$$

where *N* is the total number of decoder stages to which objectness heads are attached.

This loss guides the network to accurately identify object-like regions, complementing the pixel-wise semantic segmentation objective and improving generalization to out-of-distribution anomalies.

#### Boundary-Aware Contrastive Loss (BAC Loss)

To improve the spatial localization accuracy, especially around object edges and transition regions, we introduce a Boundary-Aware Contrastive Loss (BAC Loss) that contributes to the discriminative feature learning at semantic boundaries. While existing pixel-wise losses such as cross-entropy and Dice focus on region-level consistency, they often result in blurry boundaries or spatial over-smoothing, which is harmful for anomaly localization.

The BAC Loss addresses this problem by ensuring feature similarity among edge pixels of the same class but dissimilarity between surrounding boundary pixels of different classes. This is accomplished by comparing pixel-level embeddings in boundary regions to their 8-connected neighbors after initially leveraging a Laplacian kernel to create a binary boundary mask from the ground truth.

The BAC Loss is defined as,

19 $$\begin{aligned} \boldsymbol{L}_{{\textbf {BAC}}} \boldsymbol{= -\frac{1}{|B|} \sum _{i \in B} \sum _{j \in \mathcal {N}(i)} \left[ \mathbb {I}_{(y_i = y_j)} \log \sigma \left( \frac{f_i \cdot f_j}{\tau } \right) + \mathbb {I}_{(y_i \ne y_j)} \log \left( 1 - \sigma \left( \frac{f_i \cdot f_j}{\tau } \right) \right) \right] } \end{aligned}$$

Where, *B* is the set of boundary pixels, $\mathcal {N}(i)$ denotes the 8-connected neighbors of pixel *i*, $f_i,f_j \in \mathbb {R}^{C}$ are the feature vectors are extracted from the final decoder layer, $y_i,y_j$ are the corresponding ground-truth labels, $\tau$ is a temperature hyperparameter (set to 0.1), $\sigma$ is the sigmoid function, and $\mathbb {I}$ is the indicator function.

**Fig. 7:**
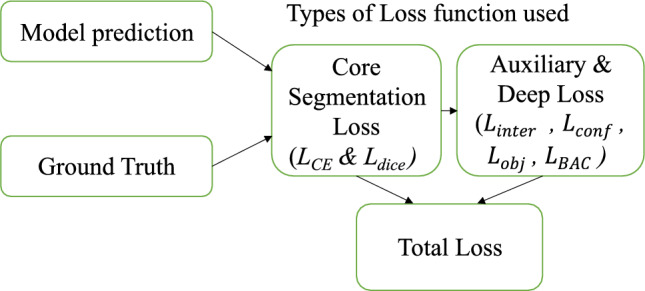
Types of Loss functions.

This contrastive supervision selectively enhances boundary precision while maintaining global coherence. We assign a relatively low weight $(\lambda _{BAC} = 0.01)$ to balance its effect within the total loss. The Fig. 7 shows the various types of loss functions used in this method.

The total training loss is the weighted sum of all components

20 $$\begin{aligned} \boldsymbol{L}_{{\textbf {total}}} \boldsymbol{= L}_{{\textbf {seg}}} \boldsymbol{+ L}_{{\textbf {inter}}} \boldsymbol{+ \lambda }_{{\textbf {conf}}} \boldsymbol{L}_{{\textbf {conf}}}\boldsymbol{ + \gamma }_{{\textbf {obj}}} \boldsymbol{L}_{{\textbf {obj}}} \boldsymbol{+ \lambda }_{{\textbf {BAC}}} \boldsymbol{L}_{{\textbf {BAC}}} \end{aligned}$$

Where $\lambda _{conf}$ and $\gamma _{obj}$ are hyperparameters that control the influence of the confidence and objectness regularization terms, respectively. we use $\lambda _{conf}=1.0$, $\gamma _{obj} = 0.2$ to ensure stable joint optimization and $\lambda _{BAC}=0.01$ to ensure subtle but effective supervision at semantic edges.

## Experimental design and comprehensive evaluation

### Experimental setup

#### Dataset preparation

Primary Training Dataset – Cityscapes Our segmentation model primarily utilizes the Cityscapes dataset for training the model. The dataset consists of 2, 975 images with a high spatial resolution of $1024\times 2048$ pixels, comprising 19 semantic classes representing urban street scenes. Images of 50 different cities in Germany and surrounding European areas have been captured to provide a wide range of scenes with varying geographical, temporal, and environmental characteristics. The annotations provide detailed pixel-level labeling for objects like roads, pedestrians, vehicles, traffic signs, and vegetation. The dataset provides both finely annotated labels, intended for training and validation. In addition, the data comprises a range of driving conditions, including various weather situations (clear, rainy, foggy), seasons (summer to winter), and different times of day (morning to dusk). Cityscapes are particularly well-suited for learning priors of urban scenes, especially in real-time semantic segmentation applications for autonomous driving and smart city environments. Evaluation Datasets – RoadAnomaly21 We assess the robustness of our model in out-of-distribution scenarios by using the RoadAnomaly21 dataset, which consists of 100 high-resolution images depicting typical urban driving environments with anomalous or unknown objects that are not found in standard training datasets such as Cityscapes. Pixel level annotations were used to identify foreign objects such as animals, debris, fallen trees and other anomalous elements of roadside infrastructure in complex visual scenes with occlusions, motion blur and low illumination. In line with the official evaluation procedure of SegmentMeIfYouCan^40^, we used a Cityscapes trained model in zero-shot setup without any fine tuning on the RoadAnomaly21 dataset. To provide a comprehensive benchmark for selective prediction and generalization performance of models under out of distribution, anomaly-aware metrics including FPR@95TPR, AUROC and overall IoU were used to evaluate the model.

#### Preprocessing pipeline

1. **Spatial Normalization:** Since we consider inference as the primary goal and aim to reduce the computational costs, the images were resized into 224 x 224.
2. **Intensity Normalization:** Per-channel normalization using ImageNet statistics (mean= [0.485, 0.456,0.406], std= [0.229, 0.224, 0.225])
3. **Data Augmentation Strategy**
  Random horizontal flipping ($p=0.5$)
  Random scaling (factor range: 0.5–2.0)
  Random rotation ($\pm 10^\circ$ to preserve road geometry)
  Color jittering (brightness $\pm 0.2$, contrast $\pm 0.2$, saturation $\pm 0.2$, hue $\pm 0.1$)
  Random erasing ($p=0.1$, scale = 0.02–0.33) for improved robustness
  Gaussian noise injection ($\sigma =0.01$) for sensor noise simulation

#### Training configuration and hyperparameters

The proposed LightMask architecture was developed in PyTorch 2.0.1 with CUDA 12.1 support for GPU acceleration (on an NVIDIA RTX 4000 Ada generation). Resized RGB images (224 × 224 pixels) were used for training and testing. Images were normalized to follow ImageNet image statistics. Optimization of the training process was carefully managed to find a balance between rapid convergence and good generalization performance. The Adam optimizer was used to train the model, with its hyperparameters set as follows: $\beta _1 = 0.9,\ \beta _2 = 0.999,\ \text {and}\ \epsilon = 10^{-6}$, with a weight decay of $10^{-5}$, excluding biases and batch normalization layers. Also, a cosine annealed learning rate schedule with warm restarts ($T_0=10, T_{mult}=2$), where the initial learning rate was increased from $10^{-5} \rightarrow 10^{-4}$ during the first 5 epochs linearly. Then, followed by decreasing it to a minimum value of $10^{-7}$ with a maximum learning rate of $10^{-4}$. To prevent large gradients that can cause training instability, we employed a gradient clipping technique that limited the maximum norm of the gradient to 1.0. Then, our model uses effective batch size 32 where we employed gradient accumulation over 8 iterations (with a base batch size 4). Training was conducted for up to 200 epochs, with an early stop criterion activated when there was no improvement in the validation error for 15 consecutive epochs.

### Comprehensive evaluation metrics results

We utilize a range of quantitative metrics that are commonly used in the fields of anomaly segmentation and open-set semantic segmentation^36^.

1. **Classification Metrics**
  - **Precision:** Measures the proportion of correctly identified anomalous pixels among all predicted anomalies.
    21 $$\begin{aligned} {\textbf {Precision}} \boldsymbol{=} \frac{{\textbf {TP}}}{{\textbf {TP}} \boldsymbol{+} {\textbf {FP}}} \end{aligned}$$
  - **Recall:** Measures the ability to capture all true anomalous regions.
    22 $$\begin{aligned} {\textbf {Recall}} \boldsymbol{=} \frac{{\textbf {TP}}}{{\textbf {TP}} \boldsymbol{+} {\textbf {FN}}} \end{aligned}$$
  - **F1 - Score:** Harmonic mean of precision and recall, used to balance the trade-off between detection accuracy and coverage, especially in imbalanced anomaly segmentation tasks.
    23 $$\begin{aligned} \boldsymbol{F}_{\boldsymbol{1}} \boldsymbol{=} \frac{\boldsymbol{2 \times } {\textbf {Precision}} \boldsymbol{\times } {\textbf {Recall}}}{{\textbf {Precision}} \boldsymbol{+} {\textbf {Recall}}} \end{aligned}$$
2. **Spatial Overlap Metrics**
  - **Intersection over Union (IoU):** Evaluates the spatial overlap between predicted and ground-truth anomaly regions.
    24 $$\begin{aligned} {\textbf {IoU}} \boldsymbol{=} \frac{\boldsymbol{|A \cap B|}}{\boldsymbol{|A \cup B|}} \end{aligned}$$
  - **Dice Coefficient:** Alternative region-based overlap metric, emphasizing symmetric similarity.
    25 $$\begin{aligned} {\textbf {Dice}} \boldsymbol{=} \frac{\boldsymbol{2|A \cap B|}}{\boldsymbol{|A| + |B|}} \end{aligned}$$

The LightMask model produces real time inference at a rate of 136.5 frames per second using only 4.29 million parameters (16.35 MB). Therefore, it was capable of providing a good balance between efficiency and quality of segmentation, as shown in Table 1. The architecture is built around an EfficientNet backbone to extract features, SSA modules to model multi scale contexts, a progressive decoder with early termination, and an objectness head to perform fine grained anomaly segmentation. Therefore, it provides a good trade-off between performance and computation cost, suitable for smart city applications.

**Table 1:** Architectural overview of LightMask.

**Model Component**	**Param #**
Backbone: EfficientNet	4,007,548
Attention Modules	138,773
ProgressiveDecoder	127,827
AdaptiveInference	929
ObjectnessHead	10,465
**Total Parameters:** 4,285,580	
**Trainable Parameters:** 4,285,580	
**Model Size (FP32):** 16.35 MB	

**Fig. 8:**
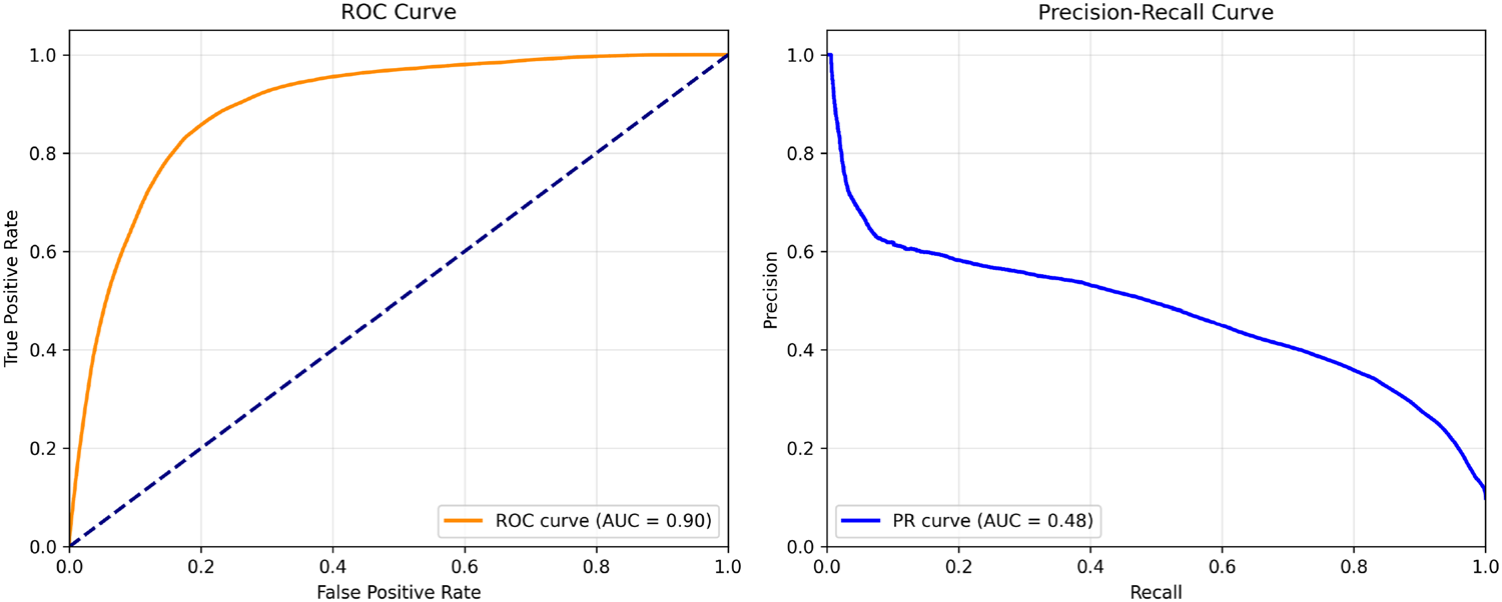
ROC (Left) and Precision-Recall curves (Right) for LightMask on Road Anomaly dataset.

As depicted in Fig. 8 the ROC curve has achieved an AUC of 0.90, which indicates that the model is able to keep a relatively high True Positive Rate at different thresholds of decision. Similarly, the AUC of the Precision Recall Curve 0.48, as demonstrated by the substantial class bias between anomalies and normal background pixels, also validates the efficiency of the model to isolate rare abnormal pixel elements from the majority normal background. The performance metrics support the model’s viability to provide reasonable sensitivity/precision trade-off values for the segmentation of anomalies in a variety of real-world applications, where operational thresholds are likely to be determined by application-specific requirements.

**Fig. 9:**
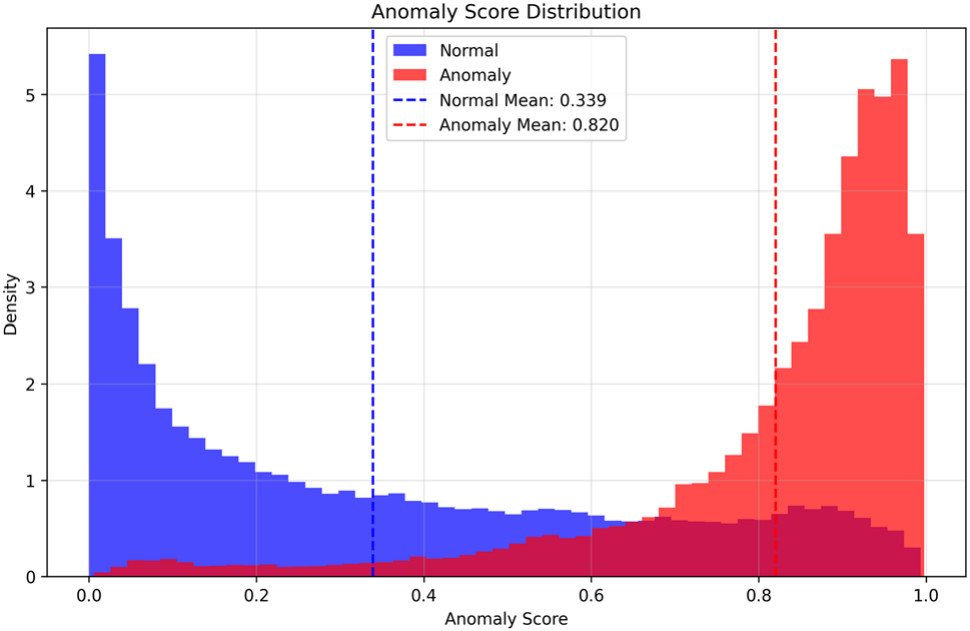
Distribution of predicted anomaly scores across normal and anomalous regions.

The anomaly scores are clearly separated for normal and anomalous images as shown by Fig. 9. Anomalous areas are concentrated on the right side of the score histogram, while normal area values are located on the left side of the score histogram. This confirms the discrimination ability of the objectness-aware prediction head of LightMask. It also demonstrates that it can be used effectively for threshold-based decision making and outlier segmentation. The large difference between the highest possible and lowest possible score results in accurate localizing of anomalies and very few false alarms.

**Table 2:** Detailed classification performance of LightMask segmented by class.

**Class**	**Precision**	**Recall**	**F1-Score**	**Support**
Normal	0.99	0.70	0.82	2,731,954
Anomaly	0.25	0.93	0.40	296,606
Accuracy	–	–	0.72	3,010,560
Macro Average	0.62	0.81	0.61	3,010,560
Weighted Average	0.92	0.72	0.78	3,010,560

As shown in Table 2, LightMask achieves 93% of all anomaly pixel detections and 99% of all normal pixel detections. These results show that LightMask can perform well when there is an extreme imbalance in the number of samples from each of the classes. The macro average F1-score for anomaly detection using LightMask was 61%. A weighted average F1-score of 78% was achieved for background classification. The computational complexity of LightMask is evaluated through floating point operation counts (FLOPs) which is 8.72 GFLOPs. Therefore, LightMask has sufficient computational resources to support many smart city applications.

**Fig. 10:**
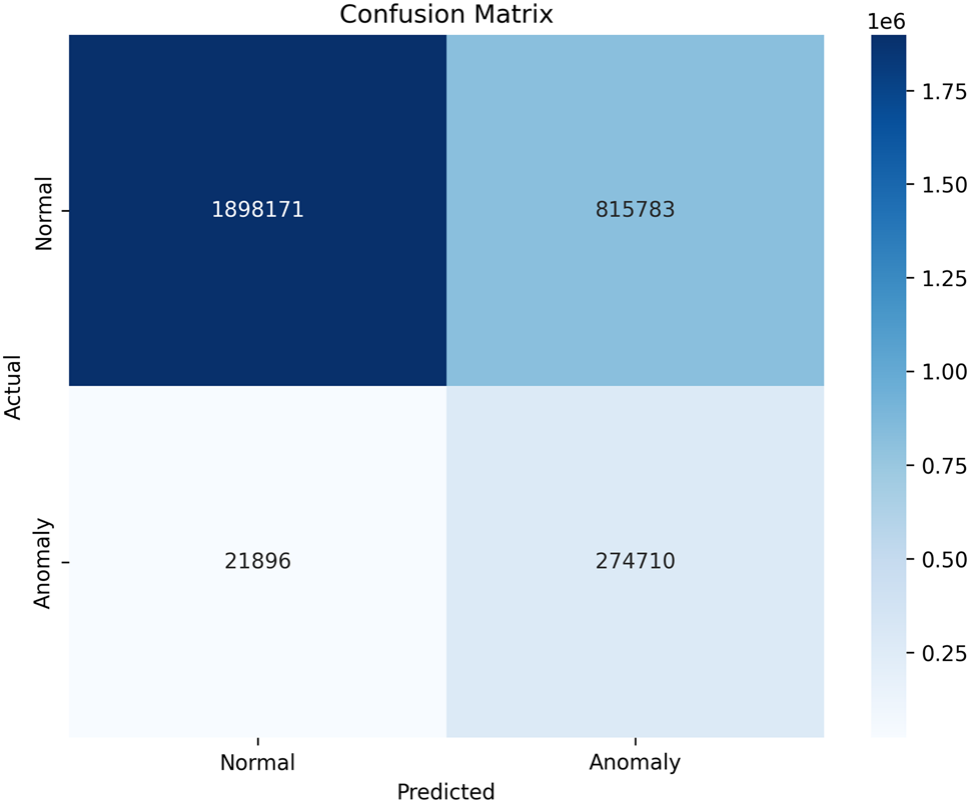
Pixel-level Confusion matrix for normal and anomaly classes.

Classification distribution between normal and anomalies was presented in Fig. 10, where it is demonstrated that the model can accurately identify an anomalous pixel as such with a recall rate of 93 %. Additionally, the model also achieves a good level of accuracy when classifying normal areas at 70 %. Thus, this analysis demonstrates the conservative nature of the model, which prioritizes recalling anomalous pixels over misclassifying normal ones in order to avoid a false negative in urban applications.

As the Comprehensive evaluation of the model gives an accuracy of 72.18% and a precision of 91.79%, makes the model a robust discriminator between anomalous and normal areas. A recall of 72.18% and an F1 score 77.66% provide additional evidence of a balanced performance in an open set environment. LightMask achieves AUC-ROC of 88.28% AUC-PR of 44.72% which shows resiliency against class imbalance and high pixel-wise distinction of anomalies. The model has a low false positive rate when the (true positive rate) TPR 95% (false positive rate) FPR@95 36.24%, and mIoU 24.68%, which, not only represents the spatial coherence of the segmented anomalies. It is expresses that the system offers real-time inference speed up to 136.5 FPS with a minimal computational overhead which is expressed as a final loss of 0.3134. Finally confirming its suitability for high-throughput deployment in running environment corresponding to safety-critical smart cities.

### Comparison with state-of-the-art methods

**Table 3:** Quantitative Comparison with Existing Models on Cityscape.

**S. No**	**Method**	**Backbone**	**Params (M)**	**mIoU**	**FPS**	**FLOPs (G)**
1	SegFormer^24^	MiT-B5	84.7	84	2.5	1447.6
2	DeepLabV3+^28^	ResNet-101	62.7	80.9	1.2	2032.3
3	PSPNet^29^	ResNet-101	68.1	78.5	1.2	2048.9
4	RTLinearFormer^25^	ImageNet	23.96	78.41	66.7	117.68
5	FCN^41^	ResNet-101	68.6	76.6	1.2	2203.3
6	DeepLabV3+^42^	MobileNetV2	-	72.8	40.7	-
7	SegNet^30^	Encoder-decoder	29.45	72.8	23.4	-
8	EfficientNet^32^	EfficientFormerV2-L	26.1	45.2	-	-
9	**LightMask (Ours)**	**EfficientNet-B0**	**4.29**	**24.68**	**136.5**	**8.72**

Our proposed LightMask model represents a significant advancement in lightweight open-set segmentation with less computational overhead makes the model suitable for smart city applications. With only 4.29 million parameters and 8.72 GFLOPs, LightMask achieves the lowest computational footprint among all evaluated models, utilizing approximately 20× fewer parameters than SegFormer and more than 1000× fewer FLOPs (8.72 GFLOPs vs. 744.8 GFLOPs). Compared to RTFormer variants, it consumes over 100× fewer FLOPs, making it suitable for safety critical applications. LightMask also demonstrates exceptional inference speed at 136.5 FPS, which is 55× faster than SegFormer and nearly 2× faster than RTLinearFormer. The mIoU of 24.68% emulates the architectural prioritization of computational efficiency primarily, LightMask maintains strong detection oriented performance with 91.79% precision and 93% recall, demonstrating effective anomaly identification with low computational overhead. As shown in Table 3, LightMask promisingly demonstrates that transformer-based anomaly segmentation can operate effectively within low computational costs. In this respect, LightMask is an effective option to be applied in urban environments which require high detection reliabilities while having low computation capabilities.

**Fig. 11:**
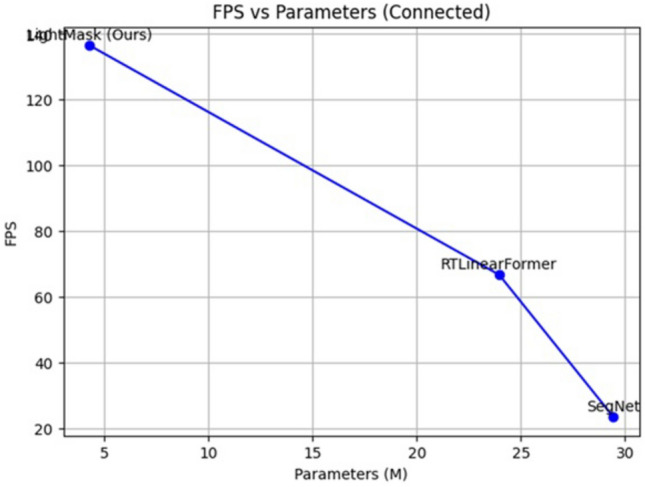
Inference speed(FPS) vs Parameter count Comparison.

As can be seen from the results shown in the above Fig. 11, LightMask (ours) achieves the highest frame rate (136.5 FPS) and reduced parameter footprint (4.29 M) compared to RTLinearFormer and SegNet. This inverse relationship between parameters and frames per second further indicates that LightMask is very efficient computationally and therefore can be used effectively in smart cities.

**Fig. 12:**
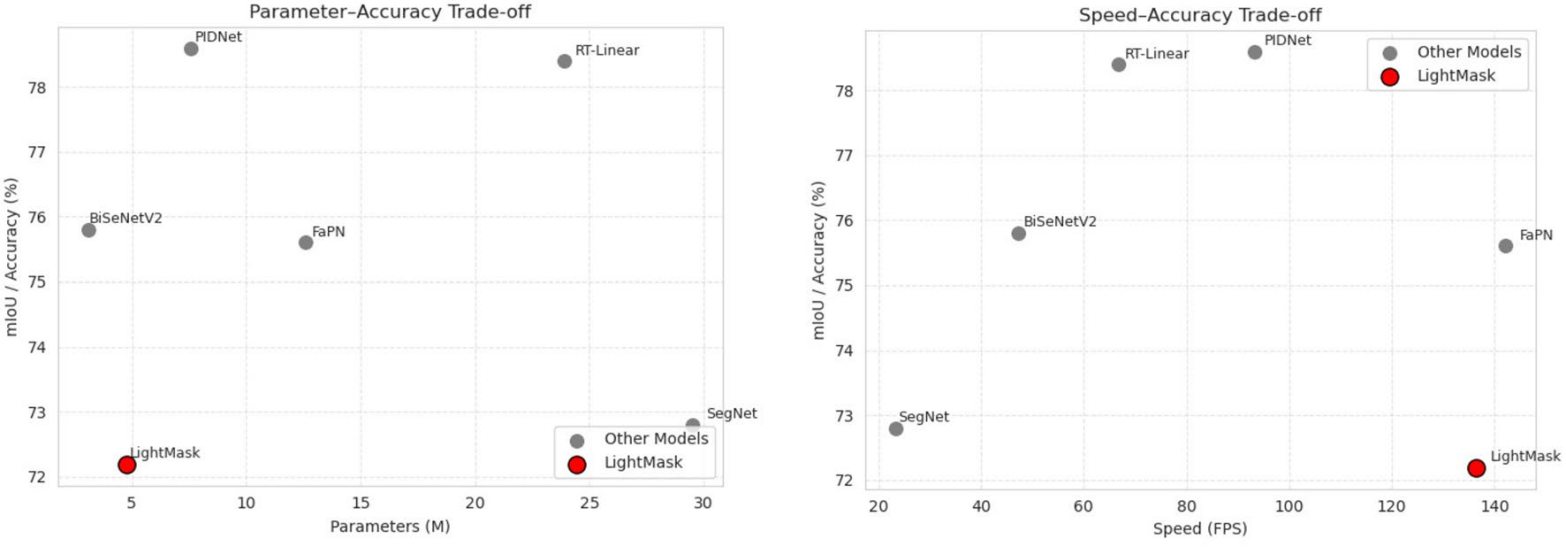
LightMask performance trade-off.

LightMask shows its efficiency with less than 5M parameters while also running at 136 FPS, it achieved 72.2% accuracy and 24.68% mIoU. The model performance has less parameter count and speed of all the other models compared to it. The models, PIDNet^43^ (with an 8M parameter count and 78.5% accuracy), and FaPN^44^ (with a 13M parameter count and 75.6% accuracy) have better accuracy, but LightMask has a competitive advantage because it makes a choice for computational efficiency and achieves the same level of speed as FaPN, with fewer parameters. This optimal positioning on the Pareto frontier in Fig. 12 makes LightMask ideally suited for smart city applications with lightweight structure and computational efficiency.

### Qualitative analysis

**Fig. 13:**
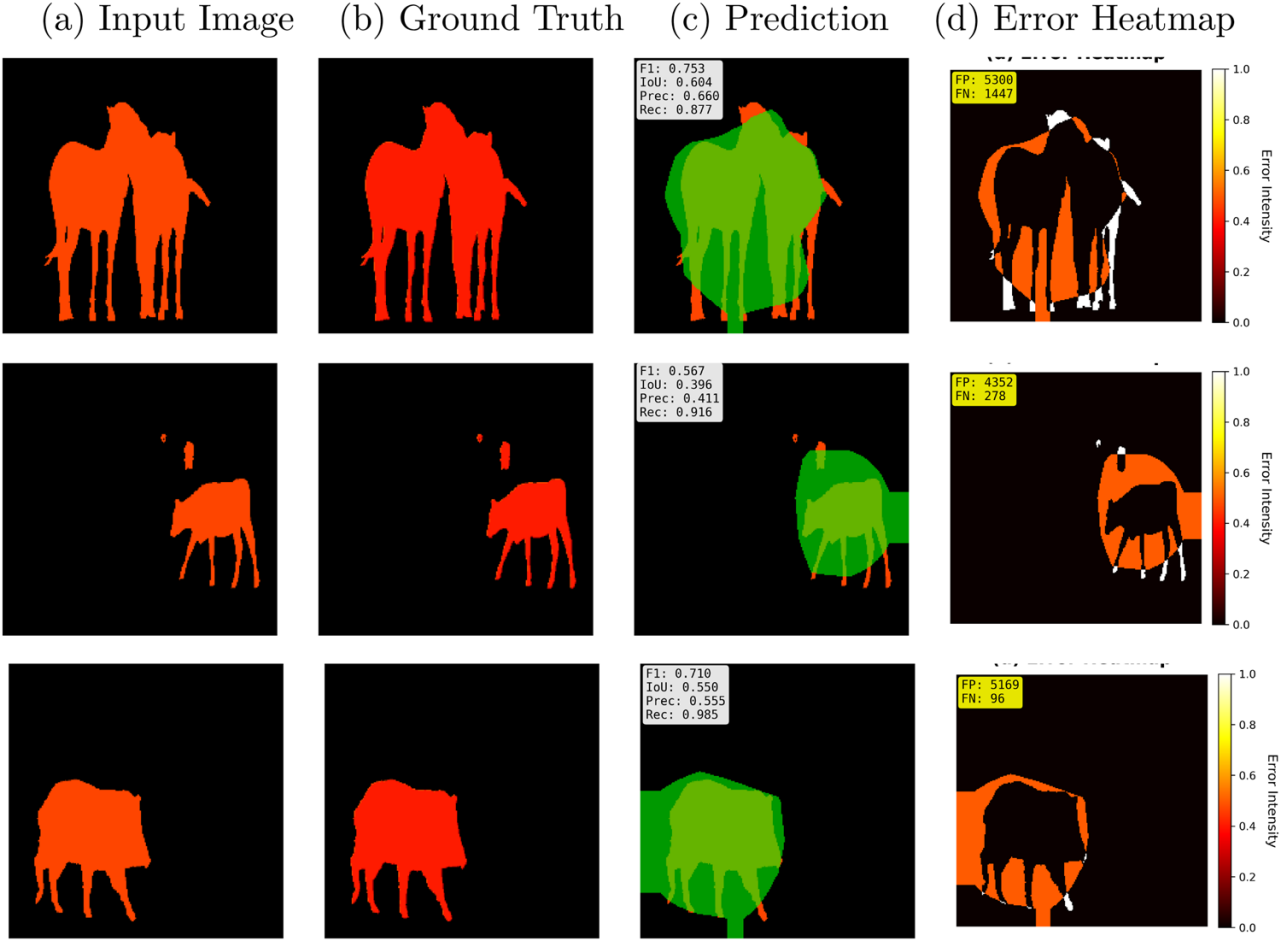
Qualitative Analysis on RoadAnomaly Dataset.

Figure 13 shows the result of qualitative analysis from the RoadAnomaly dataset samples. The four panel representation has been created with (a) input RGB image with anomalous objects, (b) Annotation of ground truth, (c) segmentation prediction, and (d) Error heatmap. The visualization depicts the color schemes, where the green color ensures correctly predicted anomalies as True Positive, the red color indicates missed anomalies as False Negatives. Normalized intensity (0.0 −1.0) has been used in the error heatmap in which white/yellow color with the high values indicates the maximum prediction error. The performance variations has been shown, in which having the FP value of 5300 confirms over segmentation. Sample with F1 0.567 and Recall 0.411 confirms the under-segmentation and the last sample highlights the highest recall value 0.985 but have higher false positive value as FP 5169. The error heatmap visualizes the object contours, confirming that LightMask has strong boundary awareness and consistent focus on structural details. These findings ensure the effectiveness of detecting anomalous objects in urban environments and also validated the quantitative results.

### Ablation study

We carry out a comprehensive ablation study to systematically evaluate the contribution of each architectural component in the proposed LightMask model. The specific design choices and their outcomes on anomaly segmentation performance was indicated with this ablation study.

**Table 4:** Component wise Ablation study.

Configuration	F1-Score	mIoU	FPS	Params (M)
Baseline (EfficientNet-B0)	0.7234	0.2145	98.3	5.30$^{1}$
+ Separable Attention	0.7410	0.2253	124.5	4.80$^{2}$
+ Progressive Decoding	0.7588	0.2325	128.9	4.50$^{3}$
+ Adaptive Complexity	0.7695	0.2402	134.1	4.40$^{4}$
+ ObjectnessHead + boundary contrastive Loss	0.7766	0.2468	136.5	4.29$^{5}$

$^{1}$Standard encoder-decoder.

$^{2}$Lightweight attention mechanism.

$^{3}$Multi-scale refinement.

$^{4}$Dynamic inference based on gradient difficulty.

$^{5}$Full architecture model.

The progressive embedding of architectural modifications into the LightMask framework gives incrementally improved performance in terms of accuracy of segmentation and computational efficiency, as clearly seen in Table 4. By successively adding separable attention, progressive multi-scale decoding, adaptive complexity estimation and auxiliary heads including region-level objectness and boundary-aware contrastive learning, the model achieves its best performance, i.e., F1-score 0.7766 and mean IoU 0.2468. Despite being designed for lightweight models, our LightMask allows a real-time inference speed of 136.5 FPS with only 4.29m parameters which shows that it can be used for smart-city applications. This good balance between prediction performance and the saving of computational overhead makes LightMask an ideal model for open-set segmentation tasks in urban environments, such as in autonomous driving, traffic monitoring, surveillance analysis and environment monitoring infrastructure.

**Table 5:** Attention Strategy Comparison.

**Attention Type**	**F1-Score**	**mIoU**	**FPS**	**Time Complexity**	**Space Complexity**
No Attention	0.7234	0.2145	98.3	O(HW)	O(HW)
Standard Self-Attention	0.7410	0.2253	67.8	O(N$^{2}$)	O(N$^{2}$)
SSA	0.7588	0.2325	124.5	O(N)	O(N)
SSA + Adaptive Gating	**0.7766**	**0.2468**	**136.5**	O(N)	O(N)

Table 5 is a comparison of several different attention mechanisms which were included in LightMask. SSA provided significant enhancements to accuracy and processing speed while retaining a linear time complexity. Additionally, the inclusion of Adaptive Gating enhanced the efficiency and performance of the system with the highest F1-score and FPS being achieved by the combination of these two techniques. These results support that lightweight, adaptable attention can be effective for anomaly segmentation.

**Table 6:** Feature Dimension Analysis.

**Feature Dimensions**	**F1-Score**	**AUC-ROC**	**AUC-PR**	**Model Size (MB)**	**FPS**
[24]	0.7234	0.8571	0.4123	12.4	145.5
[80]	0.7410	0.8654	0.4325	13.5	141.0
[192]	0.7522	0.8720	0.4391	15.2	138.3
[24, 80]	0.7639	0.8752	0.4457	15.8	137.2
[80, 192]	0.7695	0.8789	0.4462	16.1	136.8
[24, 80, 192]	0.7766	0.8828	0.4472	16.3	136.5

Table 6 assessed the impact of integrating multi-scale features within LightMask. Integrating higher dimensional features ([80], [192]) resulted in an improvement to the overall quality of the anomaly segmentation. However, the most balanced approach between accuracy and efficiency was achieved using the full fusion strategy [24, 80, 192]; as evidenced by the systems F1-score of 0.7766 and AUC-ROC of 0.8828; all while having 4.3 million parameters and 136.5 FPS, thereby enabling the system to operate in urban applications. This confirms that hierarchical feature diversity enhance the generalization in complex urban environments.

**Table 7:** Ablation Study on $\lambda _{conf}$, $\gamma _{obj}$, and $\lambda _{BAC}$.

**Configuration**	$\boldsymbol{\lambda _{conf}}$	$\boldsymbol{\gamma _{obj}}$	$\boldsymbol{\lambda _{BAC}}$	**mIoU (%)**	**Precision (%)**	**Recall (%)**	**F1 (%)**
Baseline	0	0	0	24.77	89.13	83.01	85.16
Conf_0.5	0.5	0	0	24.75	89.10	83.25	85.33
Conf_1.0	1.0	0	0	13.37	87.06	53.80	62.70
Conf_1.5	1.5	0	0	11.52	88.29	28.37	32.02
Obj_0.1	1.0	0.1	0	11.51	88.47	30.08	35.06
Obj_0.2	1.0	0.2	0	20.31	89.95	70.64	76.25
Obj_0.3	1.0	0.3	0	26.53	90.63	80.96	84.06
BAC_0.005	1.0	0.2	0.005	**37.71**	91.78	**90.50**	**90.83**
BAC_0.01	1.0	0.2	0.01	29.91	**92.36**	80.23	83.72
BAC_0.02	1.0	0.2	0.02	23.71	91.02	74.05	75.83
Full_Mode	1.0	0.2	0.01	**27.43**	**89.35**	**86.84**	**87.78**

To validate each component’s contribution in LightMask, the systematic ablation study has been conducted for evaluating the model performance at the 100 epoch checkpoint using identical training and evaluation protocols (Cityscape training and RoadAnomaly evaluation with batch size 4, EfficientNet backbone). The Table 7 represents the results of three components: confidence-weighted loss ($\lambda _{conf}$), object level attention loss ($\gamma _{obj}$) and boundary aware contrastive loss ($\lambda _{BAC}$). The baseline model shows optimal performance with higher values cause recall degradation. The object level attention progressively improves spatial coherence and the Full model achieves F1 87.78% at 100 epochs, with more traditional hyperparameter enabling stable long-term convergence. In the comprehensive evaluation at 200 epochs the model reports F1 77.66% with significantly improved precision 91.79% and AUC-ROC 88.28% demonstrating a precision with favorable convergence pattern where extended training trades sensitivity for false positive reduction is a desirable characteristics for safety critical smart city applications.

### Discussion

LightMask was limited during training and testing due to GPU memory constraint on an NVIDIA RTX 4000 Ada Generation GPU when running the image analysis at a resolution of 224x224 pixels. This resolution signifies the trade-off between computational efficiency and the level of detail that can be extracted from the images. Although it is lower than the native resolution of Cityscapes (1024x2048), we have found that for anomaly detection the resolution is sufficient to allow us to achieve a high precision (91.79%) and recall (93%). Our architecture using boundary aware contrastive loss has demonstrated an ability to extract features effectively and to delineate boundaries at this resolution. It is possible that higher resolutions could improve the spatial detail and IoU performance, but would require some architectural changes, such as adding gradient checkpoints, or training with mixed precision, or using a patch-based processing approach to better utilize available memory. The ablation study provided valuable insights into how each of the components of our architecture contribute to the overall performance during training, and highlighted boundary aware contrastive learning as the most important module contributing to our architecture’s performance. The performance has increased from 100 epochs at the end of the ablation study to 200 epochs at the end of our comprehensive evaluation, suggests a convergence pattern focused on maximizing precision by extending training to reduce false positives rather than increasing sensitivity. Together with the consistently high precision achieved in both the evaluation stages with discriminative capabilities shows 88.28% AUC-ROC and the real-time performance of 136.5 FPS, these results demonstrate that the LightMask architecture is suitable for safety-critical applications in urban environments.

## Conclusion

This paper has proposed LightMask, a lightweight transformer-based architecture for open-set anomaly segmentation in smart city applications that reduces the computational overhead with five contributions: optimized EfficientNet-B0 backbone, SSA with linear complexity, progressive decoder with dynamic early termination, adaptive inference mechanism, and auxiliary enhancements for open-set generalization. With 4.29 M parameters (16.35 MB) and 8.72 GFLOPs, the experimental results highlight the lightweight structure and computational efficiency, positioning the model as a feasible solution for smart city applications in urban infrastructure. As the results show the metrics for how well the models performed. They show 91.79% precision, 93% recall, 88.28% AUC-ROC and 36.24% FPR95, and all of these metrics indicate that the model has a high degree of robustness. In addition to demonstrating the performance of the model, the assessments were conducted using the NVIDIA RTX 4000 Ada GPU reveal that the model has an excellent segmentation capability, due to its lightweight architecture. It also has efficient computation with 136.5 FPS for performing anomaly detection in smart cities.

As the future work, the LightMask can be extended, through implementation on edge computing platforms such as Jetson, RK3588, and Raspberry Pi. This will enable further study into its deployment feasibility. The LightMask could be assessed by benchmarking on edge computing hardware, including memory profiling, latency and power consumption. Furthermore, the framework of LightMask could be extended to include multi-temporal anomaly modeling for increased contextual awareness, and to support federated learning based on distributed systems, multi-modal sensors, and mechanisms to estimate uncertainty for safety critical applications. Ultimately, the extension of LightMask to support the above said methods will enable LightMask to be used in practical deployment in multiple smart city application areas.

## Data Availability

The datasets used in this study are publicly available. The Cityscapes dataset can be accessed at https://www.cityscapes-dataset.net, and the Road Anomaly dataset is available at https://www.epfl.ch/labs/cvlab/data/road-anomaly/.
